# Effects of dairy intake on weight maintenance

**DOI:** 10.1186/1743-7075-5-28

**Published:** 2008-10-24

**Authors:** Michael B Zemel, Joseph E Donnelly, Bryan K Smith, Debra K Sullivan, Joanna Richards, Danielle Morgan-Hanusa, Matthew S Mayo, Xiaocun Sun, Galen Cook-Wiens, Bruce W Bailey, Emily L Van Walleghen, Richard A Washburn

**Affiliations:** 1 The University of Tennessee, Knoxville, TN, USA; 2 University of Kansas, Lawrence, KS, USA; 3 University of Kansas School of Medicine, Kansas City, KS, USA; 4 University of Massachusetts, Boston, MA, USA

## Abstract

**Background:**

To compare the effects of low versus recommended levels of dairy intake on weight maintenance and body composition subsequent to weight loss.

**Design and Methods:**

Two site (University of Kansas-KU; University of Tennessee-UT), 9 month, randomized trial. Weight loss was baseline to 3 months, weight maintenance was 4 to 9 months. Participants were maintained randomly assigned to low dairy (< 1 dairy serving/d) or recommended dairy (> 3 servings/d) diets for the maintenance phase. Three hundred thirty eight men and women, age: 40.3 ± 7.0 years and BMI: 34.5 ± 3.1, were randomized; Change in weight and body composition (total fat, trunk fat) from 4 to 9 months were the primary outcomes. Blood chemistry, blood pressure, resting metabolism, and respiratory quotient were secondary outcomes. Energy intake, calcium intake, dairy intake, and physical activity were measured as process evaluation.

**Results:**

During weight maintenance, there were no overall significant differences for weight or body composition between the low and recommended dairy groups. A significant site interaction occurred with the low dairy group at KU maintaining weight and body composition and the low dairy group at UT increasing weight and body fat. The recommended dairy group exhibited reductions in plasma 1,25-(OH)_2_-D while no change was observed in the low dairy group. No other differences were found for blood chemistry, blood pressure or physical activity between low and recommended dairy groups. The recommended dairy group showed significantly greater energy intake and lower respiratory quotient compared to the low dairy group.

**Conclusion:**

Weight maintenance was similar for low and recommended dairy groups. The recommended dairy group exhibited evidence of greater fat oxidation and was able to consume greater energy without greater weight gain compared to the low dairy group. Recommended levels of dairy products may be used during weight maintenance without contributing to weight gain compared to diets low in dairy products.

**Trial Registration:**

ClinicalTrials.gov NCT00686426

## Background

Almost two thirds of US adults are overweight or obese [1] and at any given time 50% are attempting to control their weight [2]. Dietary calcium appears to play a role in the regulation of energy metabolism [3], and data from several studies support an "anti-obesity" effect of dietary calcium [3]. High calcium diets attenuate adipocyte lipid accretion and weight gain during periods of over-consumption of an energy-dense diet in a rodent model of diet-induced obesity and increase lipolysis and preserve thermogenesis during energy restriction in this model, thereby increasing the loss of body weight and fat [4]. Notably, dairy sources of calcium exerted significantly greater effects in both attenuating weight and fat gain during over-feeding and accelerating weight and fat loss during energy restriction in rodents [3,4].

Recent clinical studies are consistent with these observations. Increasing dietary calcium intake from ~400 to ~1200 mg/day during constant energy restriction (-500 kcal/day) resulted in 26 and 28% increases in weight and fat loss, respectively, compared to the lower calcium intake over a 24-week period, while significantly (~2-fold) greater effects were noted when dairy foods were utilized as a calcium source [5]. Similarly, a shorter-term (12-week) study demonstrated that incorporation of sufficient yogurt into the daily diet to increase dietary calcium from ~500 to ~1100 mg/day without altering macronutrient intake during energy restriction of -500 kcal/day augmented fat loss by 61% compared to the low calcium group [6]. A six month clinical trial utilizing a mixture of dairy foods in obese African-American adults resulted in essentially similar effects on weight and fat loss in the presence of energy restriction [7]. However, a similar increase in dairy intake for six months in the absence of energy restriction did not alter body weight, but did result in 5.4% and 4.6% reductions in body fat and trunk fat, respectively, in obese African-Americans [7]. In contrast, increasing the dairy product intake of subjects consuming moderate levels of calcium (~800 mg/day) and dairy during energy restriction did not alter weight loss in a 48-week trial [8].

Retrospective, observational, and epidemiological reports, including a two-year study of normal weight women [9] and ten-year data from the CARDIA study [10] support an inverse relationship between dairy intake and adiposity. Further, recent data from the Women's Health Initiative clinical trial [11] demonstrate a modest but consistent significant attenuation of post-menopausal weight gain after three and seven years of follow-up in the calcium/vitamin D-supplemented group compared to the placebo group. Notably, results of this large randomized double-blinded placebo-controlled trial comparing the effects of 1000 mg calcium plus 400 IU vitamin D/day in 36,282 post-menopausal women (18,176 active treatment and 18,106 placebo) demonstrated benefit only in those whose baseline calcium intake was suboptimal, as treatment effects were only seen in the women with baseline calcium intakes less than 1200 mg/day. These findings also are supported by several epidemiological evaluations, including the Quebec Family Study [12], the Heritage Family Study [13] and the Tehran Lipid and Glucose study [14]. However, some secondary analyses [15] and clinical trials [8,16,17] have failed to find this relationship.

Nonetheless, the role of dietary calcium in attenuating adiposity is further supported by mechanistic data. The increase in calcitriol (1,25-dihydroxyvitamin D) elicited by low calcium diets modulates both lipogenesis and lipolysis, thereby increasing lipid filling and adiposity [3,18]; in addition, calcitriol suppresses adipocyte uncoupling protein 2 (UCP 2) expression and thereby reduces UCP 2-mediated mitochondrial fatty acid transport and oxidation [18,19] and adipocyte apoptosis [18]. Conversely, increasing dietary calcium from sub-optimal to optimal levels suppresses calcitriol levels, thereby reducing the efficiency of adipocyte lipid storage [3,18]. Increasing dietary calcium also results in increased fecal fatty acid excretion and energy loss which may also contribute to calcium's effect on adiposity [20,21]. The additional bioactivity (i.e. non-calcium mediated) of dairy foods has not been definitively identified, although the high concentration of leucine and of angiotensin converting enzyme (ACE) inhibitors in dairy may contribute to the additional effect [18,22].

Thus, multiple lines of evidence suggest a potentially important role for dietary calcium and dairy foods in the prevention and treatment of obesity. However, weight maintenance following successful weight loss is a critical component to the successful management of obesity, and although there are animal data to support the concept of calcium and dairy attenuation of weight and fat regain [23], no clinical or population data are yet available regarding the role of dairy foods in weight maintenance following successful weight loss. Accordingly, this study was performed to assess the effects of recommended versus low dairy diets on weight maintenance following a weight loss program undertaken by overweight and obese adults.

## Methods

This was a 2 site investigation located at the University of Kansas (KU) and The University of Tennessee (UT). Weight loss was from baseline (0) to 3 months followed by weight maintenance from 4 months to 9 months. To be eligible for weight maintenance, participants had to achieve the greater of a 10 kg or 10% weight loss from baseline. Participants who did not achieve this value were referred to alternative weight management programs. The primary outcomes were changes in weight and body composition during maintenance (4 months to 9 months).

### Participants

**Figure 1:**
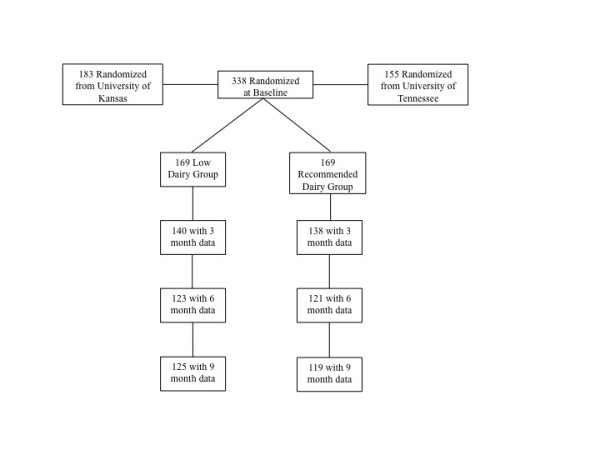
Participant flow through the study.

Potential participants underwent initial eligibility screening by telephone. Participants were 19 to 65 years of age and between 25 and 39.9 BMI. Participants were excluded if they could not participate in moderately vigorous physical activity, used special diets (i.e. vegetarian), used medications affecting metabolism (i.e. beta blockers, etc.), or were currently taking calcium supplementation. Participants were excluded if they exhibited eating disorders (score >20 on the Eating Attitudes Test) [24], restraint (score of 11 or higher on the Eating Inventory Questionnaire) [25], depression (i.e., score >35 on the Center for Epidemiological Studies Depression Scale) [26], or drug addiction (medical history). In addition, participants were excluded if they had any metabolic disease that affected energy balance (e.g. diabetes mellitus or hypothyroidism). Participants were randomized to either recommended dairy (= 3 servings/d) [27] or low dairy (= 1 serving/d) at baseline; however, the assignment was blinded to both participant and staff for 0–3 months to diminish any bias from group assignment during weight loss. A summary of randomization and participation is shown in Figure 1.

### Weight management clinics

Weight management clinics were conducted weekly for the entire investigation for ~60 minutes for both recommended and low dairy groups. The same health educator at each site led both recommended and low dairy groups to diminish investigator bias. Groups met on separate days to minimize contamination. Both groups received the same behaviorally based clinic on topics of lifestyle change, physical activity, and nutrition. For example, topics included preparation of meals, shopping, label reading, addition of fruits and vegetables (F/V), physical activity (PA), goal-setting, self-monitoring, social support, etc. Information differed only on the topic of nutrition during weight maintenance where the group randomized to recommended dairy received information and strategies for achieving = 3 servings of dairy per day and the group randomized to low dairy did not receive this information. At each meeting, participants reported the estimated energy expended each week through PA as well as the number of steps recorded by step counter. For quality assurance, clinics were supervised by one of the investigators.

### Weight loss diet (Months 1–3)

Energy intake was reduced to ~1,200 to 1,500 kcal/day using a combination of meal plans, pre-packaged meals (PMs), F/V, and shakes. A typical daily weight loss diet can be found in Table 1. Non-caloric beverages such as water, diet soda, coffee, etc. were allowed ad libitum. If participants reported hunger during the diet, they were encouraged to consume more F/V.

**Table 1:** Typical daily intake during weight loss phase

*Breakfast*		
Smoothie made with vanilla shake	<300 kcal	1 PM

1 cup berries and 1 banana		2 fruits

		

*Mid-morning snack*		

Apple	≈ 70 kcal	1 fruit

		

*Lunch*		

Vegetable Stew with Beef entrée	<300 kcal	1 PM/diet plan meal

1 cup cooked bell peppers and 1 cup diced tomatoes		2 vegetables

		

*Afternoon snack*		

Chocolate shake	≈ 100 kcal	1 PM

		

*Dinner*		

3 oz grilled skinless chicken breast 1 medium baked potato	<350 kcal	1 PM/diet plan meal

1 cup cooked broccoli		1 vegetable

1 bunch grapes		1/2 fruit

		

*Late-night snack*		

Chocolate pudding	≈ 150 kcal	1 PM/diet plan snack

1/2 sliced banana		1/2 fruit

### Weight maintenance diet (Months 4–9)

Participants consumed a weight maintenance diet using healthy eating strategies learned during clinics (i.e., portion control, record keeping, high-volume/low energy foods). Maintenance levels of calorie intake were estimated from predicted resting metabolic rate (RMR) and level of reported physical activity. Participants were encouraged to continue to use meal plans, PMs, 5 servings of F/V, and non-caloric beverages of choice as part of their weight maintenance strategy. Participants in the recommended dairy group were instructed to consume at least 3 servings of dairy per day as fluid milk, yogurt, or cheese and those in the low dairy group were instructed to consume 1 or fewer servings of dairy per day. Dairy servings were standardized at 1 cup (240 mL) milk, 1 cup (227 g) yogurt or 42 g hard cheese (e.g. cheddar).

### Moderately vigorous physical activity (MVPA)

A progressive program of MVPA (walking) was designed to target ~10,000 steps per day by week 4. Participants were given pedometers at baseline and reported steps at weekly meetings. The number of steps per day was averaged across 3 month intervals and recorded at 3, 6, and 9 months.

### Energy and dairy intake

Three day food records were obtained at baseline, 3, 6, and 9 months. The records were reviewed and clarified in an interview with a registered dietitian utilizing food models and neutral probing questions, as previously described [5,28]. Subsequently, the food records were coded and entered into a computer software system for analysis of nutrient composition, energy intake, and servings of dairy products using the USDA National Nutrient Data Base for Standard Reference .

### Staff training and site coordination

Staff training occurred at each site according to mutually approved protocols. Prior to data collection, all staff had to be certified per procedure. For example, values for dual energy X-ray absorptiometry (DEXA), food records, etc., were compared to known standards or to values generated by the investigators. Adherence to protocols was monitored by the site PI. All data collected from both sites were transferred to a data coordinator at KU to check for accuracy, completeness, outliers, etc. Data were subsequently transferred to the study statistician (MM) for analysis.

### Laboratory Procedures

All laboratory assessments were obtained at baseline, 3, 6, and 9 months using standardized protocols.

#### Height and weight

Height and weight were obtained after a 12 hour fast between the hours of 6 am and 10 am. Height was obtained using a stadiometer and weight was obtained with a calibrated scale accurate to + 0.1 kg. Participants were measured in a standard hospital gown after attempting to void.

#### Body composition and regional adiposity

DEXA (Lunar Corp.) was used to determine, total fat mass, trunk fat mass, fat free mass, and percent body fat. Women received a pregnancy test prior to each DEXA test. The DEXA was calibrated daily using the calibration block supplied by the manufacturer and weekly using a spine phantom, and each site utilized a single operator. A drift of more than 3% was established *a priori *as the action level requiring instrument service and recalibration; however, this action was not required during the study. Application software provided by the manufacturer was utilized to quantitate body composition. As a surrogate measure of abdominal adiposity, waist circumference was measured using the procedures of Lohman et al. [29].

#### Metabolic profile

Blood samples were obtained after a 12 hour fast. Serum cholesterol and triglyceride concentrations were measured by the hospital clinical laboratories at each site using an automated analyzer (Du Pont Co), and using standard enzymatic techniques. High-density lipoproteins (HDL) were measured after removal of very-low-density lipoproteins (VLDL) and low-density lipoproteins (LDL) from samples by precipitation with phosphotungstate [30]. Glucose was measured using an autoanalyzer (Beckman) and insulin was measured using a double-label antibody technique [31]. Calcitonin (Immutopics, San Clemente, CA), 25-OH-D and 1,25-(OH)_2_-D (Alpco Diagnostics, Windham, NH) levels were measured via immunoassay using commercial kits.

#### Blood pressure

Blood pressure was measured using a sphygmomanometer with the subject seated for a minimum of 5 minutes in an isolated room with the arm bared, supported, and positioned at the heart level. A cuff was selected based on measurement of the length and circumference of the arm [32]. Systolic (SBP) and diastolic pressures (DBP) were recorded [32]. Two measures were averaged and additional measures were obtained if the measures differed by more than 5 mm Hg [34,35].

#### Resting metabolic rate (RMR) and respiratory quotient (RQ)

RMR was determined by indirect calorimetry using the open circuit technique between the hours of 6 am and 10 am after a 12 hour fast and 48 hour abstention from exercise [36]. The participant rested quietly for 30 minutes in an isolated room with the temperature controlled to 21–24 degrees centigrade. Subsequently, the participant was placed in a ventilated hood for a minimum of 30 minutes. Criteria for a valid metabolic rate was a minimum of 15 minutes of steady state with steady state determined as less than 10% fluctuation in minute ventilation and oxygen consumption and less than 5% fluctuation in respiratory quotient (Sensormedics Corporation, Yorba Linda, CA). Metabolic rate was calculated using the Weir equations [37]. RQ was calculated as carbon dioxide production/oxygen consumption [38].

### Statistics and Data Analysis

Descriptive statistics for all variables were calculated for the entire study population and also calculated by treatment group at all four time points. Frequencies and percentages were used to summarize categorical variables and means and standard deviations were used to summarize quantitative variables.

The primary method of analysis was linear mixed models and the primary outcomes were change from the start of the maintenance period (3 months) to the midpoint (6 months) and end (9 months) of maintenance periods (i.e. 6 months value – 3 months value and 9 months value – 3 months value). The models had a mean structure consisting of an intercept and the following variables: baseline (time 0) value, time period (6 months, 9 months), treatment group (recommended dairy, low dairy), site (UT, KU), and an interaction of treatment group by site. Interaction between treatment group and time period was tested but was not significant in any of the models so it was not used.

The covariance structure used for the models was compound symmetric and its parameters were allowed to differ for the two treatment groups. The p-values shown in the tables for "Type III Tests of Fixed Effects" are from F-tests against the null hypothesis that the parameter value for each variable is zero given that all other variables and the interaction are in the model. Kenward-Roger adjustments for the denominator degrees of freedom were used in all models. Due to the significant site by treatment group interaction in the models, post-hoc Tukey adjusted t-tests for pair-wise comparisons of interest were calculated when the overall tests were significant. A separate model was run for each variable.

Differences within treatment groups in the mean change in weight, RMR, RQ, BMI, total body fat, trunk fat and waist circumference between baseline and 3 months was tested via a Satterthwaite adjusted two sample t-test. The same type of test was used for testing differences in dietary/energy intake variables between treatment groups at each of the four time periods. All analyses were done using SAS version 9.1 (SAS Institute Inc., Cary, NC, USA).

Registration: ClinicalTrials.gov NCT00686426

## Results

### Participants

Three hundred thirty eight participants were randomized at baseline and 270 (82%) were eligible for evaluation during maintenance. Of those not eligible for evaluation, 46 did not meet the weight loss requirements. Seventy nine percent of participants were Caucasian, 15% Hispanic, 5% African American, and 1% Other. No significant differences at baseline existed for participants in the recommended or low dairy groups (Table 2), and there were no differences between the dropouts and those remaining in the study. However, there were site differences in baseline weight and adiposity among those subjects who were subsequently eligible to enter the weight maintenance phase (Table 3). The UT site exhibited a lower body weight (97.1 vs. 102.2 kg, p = 0.0012) and fat (43.9 vs. 46.7%, p = 0.0021) compared to the KU site.

**Table 2:** Baseline characteristics by group (month 0)

	LD	RD
Age (years)	40.7 ± 6.8	39.9 ± 7.2

Weight (kg)	99.5 ± 12.9	101.1 ± 12.8

BMI (kg/m^2^)	34.6 ± 3.1	34.4 ± 3.2

Body Fat (%)	45.9 ± 7.4	45.4 ± 7.2

Waist Circumference (cm)	101.6 ± 9.2	102.7 ± 9.6

Systolic Blood Pressure (mmHg)	125.2 ± 13.0	125.4 ± 12.8

Diastolic Blood Pressure (mmHg)	82.1 ± 8.5	83.2 ± 9.1

Total Cholesterol (mg/dl)	201.3 ± 37.1	202.7 ± 35.1

HDL-Cholesterol (mg/dl)	52.7 ± 14.3	50.2 ± 12.3

LDL-Cholesterol (mg/dl)	118.5 ± 30.6	121.4 ± 31.4

Triglycerides (mg/dl)	150.5 ± 88.6	154.1 ± 88.3

Glucose (mg/dl)	94.0 ± 20.1	92.1 ± 8.8

Insulin (mcgIU/ml)	10.6 ± 7.2	10.3 ± 5.0

Values are means ± standard deviation. LD = low dairy; RD = recommended dairy. There were no significant differences between groups for any variable.

**Table 3:** Measurements for all subjects who were eligible for weight maintenance by site at baseline (month 0)

	Baseline (Month 0)		P-value for site difference
	KU (n = 163)	UT (n = 107)	

Age (years)	40.8 ± 7.0	40.0 ± 6.7	0.2464

Weight (kg)	102.2 ± 13.2	97.1 ± 11.4	0.0012

BMI (kg/m^2^)	34.7 ± 3.3	33.9 ± 2.7	0.0527

Body Fat (%)	46.7 ± 7.6	43.9 ± 6.3	0.0021

Waist Circumference (cm)	101.5 ± 9.6	103.1 ± 8.9	0.1621

Systolic Blood Pressure (mmHg)	125.7 ± 13.1	123.9 ± 12.1	0.2502

Diastolic Blood Pressure (mmHg)	82.6 ± 8.8	82.5 ± 8.4	0.9255

Total Cholesterol (mg/dl)	203.1 ± 35.1	202.1 ± 37.7	0.8245

HDL-Cholesterol (mg/dl)	51.7 ± 12.9	51.9 ± 13.9	0.9324

LDL-Cholesterol (mg/dl)	120.9 ± 28.9	119.5 ± 33.9	0.7149

Triglycerides (mg/dl)	149.7 ± 83.3	153.6 ± 93.0	0.7179

Glucose (mg/dl)	92.3 ± 18.6	93.9 ± 9.0	0.4047

Insulin (mcgIU/ml)	9.6 ± 5.6	10.6 ± 3.8	0.1254

Body Fat (kg)	44.6 ± 8.7	42.5 ± 7.3	0.0442

Trunk Fat (kg)	23.3 ± 4.4	23.0 ± 4.0	0.5891

Values are means +SD

### Energy intake during weight loss and maintenance

**Figure 2:**
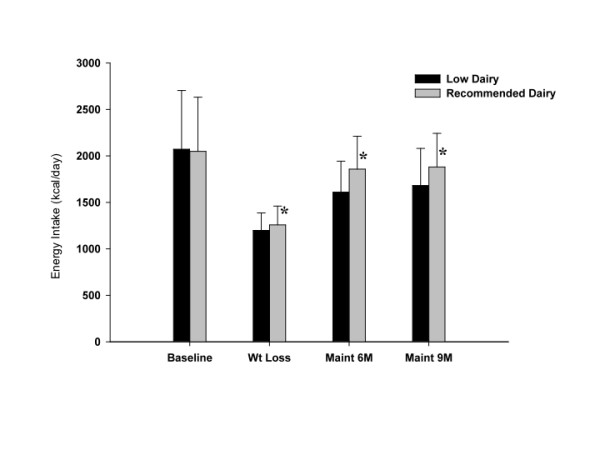
**Daily energy intake by group and time**. Values are means ± standard deviation. * Significant between group differences (p < 0.05).

Reported energy intake during weight loss was 1258 ± 202 kcal/d for the recommended dairy group and 1199 ± 187 kcal/d for the low dairy group (p = 0.01). Reported energy intake during weight maintenance at 6 months was 9% below study baseline values for the recommended dairy group and 22% below baseline values for the low dairy group. Reported energy intake during weight maintenance at 9 months remained 9% below baseline values for the recommended dairy group and was 19% below baseline values for the low dairy group. Energy intake was significantly greater (p < 0.0001) for the recommended dairy group compared to the low dairy group during weight maintenance (Figure 2). There were significant site differences in energy intake at each measured time point, with participants at the UT site consistently reporting ~100 kcal less/day than those at the KU site (Table 4); however, the aforementioned observed difference between energy intake in the recommended dairy group compared to the low dairy group was consistent across the two sites.

**Table 4:** Energy intake for all subjects who were eligible for weight maintenance by site and time

	Maintenance baseline (3 Months)^1^		6 Months^2^		9 Months^3^	
	KU (n = 163)	UT (n = 107)	KU (n = 129)	UT (n = 107)	KU (n = 136)	UT (n = 107)

Energy Intake (Kcals)	1260.1 ± 201.5	1168.2 ± 169.8	1786.2 ± 427.6	1668.6 ± 248.5	1820.1 ± 459.7	1723.7 ± 285.8

^1^Site difference: p < 0.0001

^2^Site difference: p = 0.0051

^3^Site difference: p = 0.0330

Values are means + SD

### Dairy and calcium intake during weight loss and maintenance

During weight loss, the recommended dairy group consumed 1.2 ± 0.5 servings of dairy/d and low dairy consumed 1.1 ± 0.5 servings of dairy/d (NS). From 3 to 6 months, recommended dairy consumed 3.0 ± 0.6 servings of dairy/d and low dairy consumed 0.6 ± 0.3 servings of dairy/d (p < 0.0001). From 6 to 9 months, recommended dairy consumed 3.1 ± 0.5 servings of dairy/d and low dairy consumed 0.7 ± 0.4 servings of dairy/d (p < 0.0001). During weight loss, calcium intake was 731 ± 251 mg/d for the recommended dairy group compared to 707 ± 230 mg/d for the low dairy group (NS). During weight maintenance, calcium intake was 1325 ± 254 mg/d for the recommended dairy group compared to 579 ± 166 mg/d for the low dairy group (p < 0.0001).

### Physical activity (steps)

PA was nearly identical for recommended and low dairy groups. The recommended level of dairy group at 3, 6, and 9 months had 8546 ± 2008, 8754 ± 2227, and 8765 ± 2252 steps/d, respectively. The low dairy group at 3, 6, and 9 months had 8332 ± 2320, 8729 ± 2436, and 8789 ± 2320 steps/d, respectively.

### Body weight, BMI, body composition, and waist circumference

**Figure 3:**
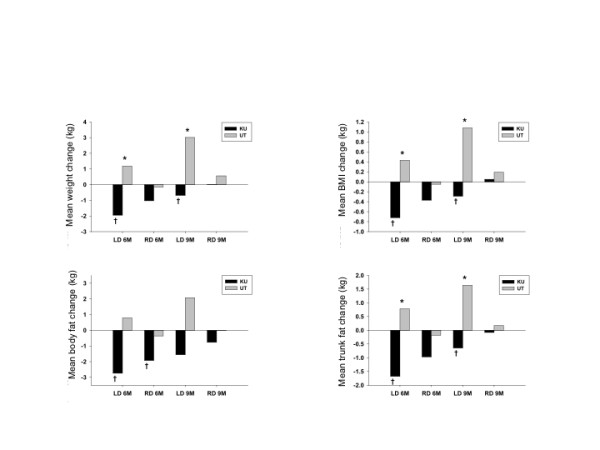
**Anthropometric changes by group and site**. Values are mean changes. KU, University of Kansas; UT, University of Tennessee; BMI, body mass index; LD = low dairy; RD = recommended dairy. * Significant between group differences for the UT site during weight maintenance (p < 0.05). † Significant between site differences during weight maintenance in the LD groups (p < 0.05).

There were no significant differences between the recommended and low dairy group for weight loss, BMI, total body fat, trunk body fat, or waist circumference from baseline to 3 months (Table 5). The primary outcomes of change in weight, BMI, body fat, and trunk fat main effects during weight maintenance showed no significant differences between the treatment groups when unadjusted for higher level interactions. After adjusting for the treatment group by site interaction the low dairy group at UT had significantly greater gains in weight, BMI, and trunk fat than the recommended dairy group at UT, and the low dairy group at UT had significantly greater gains in weight, BMI, total body fat, and trunk fat than the low dairy group at KU. Differences in the recommended dairy groups between sites were not significant, nor were differences between the recommended dairy group and low dairy group at KU. The recommended dairy groups at both sites and the low dairy group at KU exhibited weight maintenance from 4 to 9 months (Figure 3).

**Table 5:** Reductions in anthropometric measures during weight loss by group (months 0–3)

	L D	RD
Weight (kg)	11.2 ± 3.3	11.2 ± 3.8

BMI (kg/m^2^)	3.9 ± 1.1	3.8 ± 1.3

Body Fat (%)	4.6 ± 2.9	4.1 ± 3.0

Body Fat (kg)	8.5 ± 3.2	8.2 ± 3.2

Trunk Fat (kg)	4.7 ± 2.1	4.8 ± 2.1

Lean mass (kg)	2.7 ± 2.1	3.0 ± 2.3

Waist Circumference (cm)	7.9 ± 3.3	7.5 ± 4.3

Values are means ± standard deviation. LD = low dairy, RD = recommended dairy. There were no significant differences between groups for any variable.

### Metabolic profile

There were no significant differences between recommended and low dairy groups for changes in total cholesterol, triglycerides, HDL, LDL, SBP, DBP, glucose, and insulin. Both the recommended and low dairy groups showed significant increases during maintenance for cholesterol (p = 0.0001), HDL (p = 0.02), LDL (p = 0.0009), SBP (p = 0.02), and DBP (p = 0.006). Differences for triglycerides, glucose, and insulin were not significant (Table 6). There were no significant differences between recommended and low dairy groups during weight loss and maintenance for changes in 25-OH-D or calcitonin. However, during maintenance, the recommended dairy group exhibited a significant diet-induced suppression of 1,25-(OH)_2_-D. Baseline 1, 25-(OH)_2_-D was 43.8+9.8 pg/mL in the recommended dairy group and decreased by 5.8 and 6.9 pg/mL at 6 and 9 months, respectively (p < 0.0001), while there was no significant change in the low dairy group.

**Table 6:** Changes in blood pressure and bood chemistry by treatment group over time

	Baseline		3 Month		9 Month	
	LD	RD	LD	RD	LD	RD

SBP	125.2 ± 13.0	125.4 ± 12.8	117.4 ± 9.8	116.7 ± 11.3	118.8 ± 10.8*	116.9 ± 10.4*

DBP	82.1 ± 8.5	83.2 ± 9.1	77.4 ± 8.4	77.6 ± 7.8	78.4 ± 8.7*	77.9 ± 8.2*

Cholesterol	201.3 ± 37.1	202.7 ± 35.1	170.1 ± 32.5	176.0 ± 36.4	191.7 ± 34.5*	195.9 ± 34.4*

HDL	52.7 ± 14.3	50.2 ± 12.3	47.3 ± 11.2	46.8 ± 9.9	54.1 ± 12.6*	53.9 ± 12.4*

LDL	118.5 ± 30.6	121.4 ± 31.4	101.2 ± 26.7	106.6 ± 30.7	113.5 ± 30.8*	116.7 ± 32.2*

Triglycerides	150.5 ± 88.6	154.1 ± 88.3	106.1 ± 47.6	111.7 ± 47.6	119.1 ± 53.2	123.0 ± 59.2

Glucose	94.0 ± 20.1	92.1 ± 8.8	88.4 ± 7.5	88.8 ± 7.7	90.8 ± 15.1	90.0 ± 8.2

Insulin	10.6 ± 7.2	10.3 ± 5.0	6.9 ± 4.0	7.2 ± 4.5	7.8 ± 4.2	7.7 ± 4.2

Values are means ± standard deviation. LD = low dairy; RD = recommended dairy. There were no significant differences in the baseline to 3 months changes between groups. *Significant change from 3 to 9 months within treatment group (p < 0.05). There were no significant differences in change from 3 to 9 months between treatment group.

### Resting metabolic rate and respiratory quotient (RQ)

RMR declined significantly during energy restriction from baseline to 3 months for both recommended and low dairy groups (p < 0.0001). RMR showed a trend (p = 0.08) for greater increase during weight maintenance for the recommended compared to low dairy group. RQ decreased for both recommended and low dairy groups during energy restriction (p = 0.0007). During weight maintenance, the low dairy group showed a significantly greater increase (p < 0.01) in RQ compared to the recommended dairy group (Table 7).

**Table 7:** Changes in Resting Metabolic Rate (RMR) and Respiratory Quotient (RQ) by group

	Baseline		3 Month		9 Month	
	LD	RD	LD	RD	LD	RD

RMR*	1881 ± 320	1901 ± 344	1689 ± 301	1701 ± 304	1751 ± 340	1831 ± 343

RQ*†	0.77 ± 0.06	0.76 ± 0.05	0.74 ± 0.04	0.74 ± 0.05	0.77 ± 0.05	0.76 ± 0.06

Values are means ± standard deviation. LD = low dairy; RD = recommended dairy. *Significant change from baseline to 3 months (p < 0.0001). †Significant difference in the change between 3 to 9 months between groups (p < 0.01).

## Discussion

Although recommended levels of dairy have been shown to augment weight loss during energy restriction when compared to low dairy intakes, little is known about the effects of recommended levels of dairy during weight maintenance. We investigated the effects of recommended levels of dairy compared to low levels of dairy across 6 months of weight maintenance but found no differences between groups for body weight or components of body composition.

The premise of this investigation was that correction of dietary calcium and dairy insufficiency may attenuate weight regain following successful weight loss. Accordingly, it was important to have a clear comparison of inadequate versus adequate levels of intake, as supplementation of an adequate or nearly adequate diet would be predicted to exert little or no effect. For example, previous studies of the effects of dairy on adiposity during energy restriction demonstrated significant effects when the un-supplemented group had calcium and dairy food intakes of <600 mg and <1 serving/d, respectively [5-7], while increasing the dairy intake of a group consuming a more moderate level of calcium (~800 mg/d) during comparable energy restriction exerted no effect [8]. Consequently, this study was designed to distinguish between clearly inadequate levels of calcium (<600 mg/day) and dairy (<1 serving/day) with clearly adequate levels (>1,000 mg calcium and >3 dairy servings/d), and our dietary intake data demonstrate that these parameters were met.

The adherence to treatment during weight maintenance was excellent, with the recommended dairy group showing ~3 fold greater dairy intake compared to the low dairy group. This was reflected in a significant decrease in 1,25-(OH)_2_-D levels in the recommended dairy group, while no change was seen in the low dairy group. Attrition was 17.4% in the recommended dairy group compared to 14.3% in the low dairy group indicating that higher levels of dairy were well tolerated. Metabolic profile was not different between the recommended compared to the low dairy group indicating that increased consumption of dairy products did not have a negative impact on the metabolic profile.

Of interest, the low dairy group had decreased energy intake compared to the recommend dairy group at all time points yet maintenance of body weight and fat were not different. This suggests that diets with recommended levels of dairy may be higher in energy content while producing similar effects on body weight and fat as diets low in dairy. The reason for this is unclear; however, the recommended level of dairy group had a trend towards greater RMR during weight maintenance compared to the low dairy group (p = 0.08) and a significantly lower RQ (p = 0.01), indicating greater fat oxidation. Greater RMR may impact energy balance by allowing a greater energy intake for the recommended dairy group without increased weight gain when compared to the low dairy group. Additionally, the recommended dairy group also may have benefited from increased fat oxidation compared to the low dairy group, and this also may have influenced energy balance.

In support of this, calcitriol inhibits lipolysis and fat oxidation [19], and suppressing circulating calcitriol by increasing dairy food intake as observed in the present study has been reported to result in increased lipolysis in both mice [3,4,23] and humans [6,7]. Moreover, Gunther et al [39] recently demonstrated that chronic consumption (one-year) of a dairy-rich high calcium diet resulted in a significant (~two-fold) increase in post-prandial fat oxidation following a liquid meal challenge compared to subjects maintained on a low-calcium diet. Further, in a randomized controlled crossover study testing the effects of low- and high-dairy diets on substrate oxidation whole-room calorimetry Melanson et al [40] demonstrated that feeding a high dairy diet under energy deficit conditions resulted in a significant 30 g increase in 24-hour fat oxidation compared to the low dairy diet. This increase in fat oxidation represents an additional 270 kcal/day, a value in the same range as the increase in energy intake on the recommended dairy diet in the present study (248 kcal for the first half of maintenance and 200 kcal for the second half of maintenance), suggesting that an increase in self-reported energy intake in subjects consuming the recommended level of dairy may have been compensated for by a comparable increase in fat oxidation. These values also are consistent with data from Harvey-Berino et al [16]. Although they reported no effect of dairy on weight or fat loss in overweight and obese adults over a one-year period of a prescribed 500 kcal/day deficit, their data demonstrate that the high dairy group maintained an energy deficit of only 314 and 224 kcal/day at 3 and 12 months, respectively, compared to respective deficits of 442 and 402 kcal/day in the low dairy group. Thus, the high dairy group experienced comparable weight loss while consuming an additional ~150 kcal/day. In a similar randomized study, Thompson et al [8] reported similar weight loss in moderate and high dairy groups prescribed a 500 kcal/day deficit, but those consuming the high dairy diet consumed significantly more energy (~150 kcal/day) while still attaining the same weight and fat loss. Accordingly, we suggest that greater fat oxidation in the present study may have permitted a corresponding increase in energy intake in subjects consuming the recommended level of dairy intake compared to those on low dairy intakes without adversely affecting body weight or body fat.

An additional consideration is the potential direct effect of recommended vs. low levels of dairy intake on appetite and food consumption. Dairy products have been proposed to increase satiety and attenuate food intake due to both protein-induced satiety [41,42] and to the caseinomacropeptide which is released during digestion of dairy protein and stimulates cholecystokin release [43]. However, Hollis and Mattes [44] recently reported that feeding three daily dairy servings for seven days in a randomized cross-over study resulted in a significant increase in energy consumption (209 kcal/day) compared to the low dairy period. This number is remarkably consistent with the increased energy consumption by the recommended dairy group in both the present study and earlier reports [8,16]. Thus, although Hollis and Mattes reported that the additional energy provided by supplementary dairy products is not fully compensated for by a reduction in subsequent energy intake [44], these data suggest that subjects consuming recommended levels of dairy operate at a lower metabolic efficiency which may result in a shift to greater fat oxidation compared to individuals consuming low levels of dairy. Consequently, the additional energy intake does not result in corresponding gain in weight or fat. Moreover, Ochner and Lowe [45] recently reported that greater calcium intake (primarily derived from dairy sources) inversely predicted weight gain following weight loss by overweight and obese women only when controlling for energy intake. Higher energy intake significantly predicted weight regain only when controlling for changes in dietary calcium intake. Accordingly, their data indicate that weight gain from excess energy intake following weight loss is attenuated by attaining adequate levels of dairy calcium intake.

There were significant site differences with respect to weight change during the maintenance phase. During the first three months of maintenance, the low dairy group at UT exhibited weight regain that was significantly attenuated in the recommended dairy group; in contrast, the KU cohort exhibited continued weight loss during the first three months of the maintenance phase. Both patterns are common in studies of regain, with some extending adherence to behavioral strategies learned during weight loss into the early *ad libitum *dietary phase while others demonstrate rapid regain, although longer term studies consistently demonstrate regain within 12 months. Although consistent methodology was used across the two sites, it appears that there were site differences in the retention of the behavioral strategies acquired by subjects during the weight loss phase. Nonetheless, the effects of recommended dairy intakes on fat oxidation as evidenced by RQ changes and on the ability to consume more food energy than low dairy consumers without adversely affecting body weight or body composition was consistent across the two sites in both separate and combined analyses.

This study was conducted in free-living outpatients, with attendant limitations of adherence to protocol and under-reporting of energy intake. Nonetheless, key strengths of this investigation include its sample size (n = 338 enrolled, 270 completed) and adherence to diet, the latter of which is supported by confirmation of anticipated suppression of 1,25-(OH)_2_-D in the recommended dairy group during the maintenance phase.

## Conclusion

Fifty percent of adults attempt to lose and maintain weight loss annually. Individuals frequently utilize diets that restrict nutrients and eliminate food groups in the effort to achieve weight loss and maintenance, and dairy products are often viewed as potentially fattening by many who diet, especially women. This investigation could find no disadvantage for weight maintenance by consuming a diet with the recommended levels of dairy products compared to a low level of dairy products. Those who did consume the recommended amount of dairy products during weight maintenance exhibited evidence of greater fat oxidation and were able to consume a greater amount of total energy compared to those who consumed less dairy products without any additional weight gain. Being able to consume greater amounts of energy may provide benefit for chronic adherence to a weight maintenance diet. This study indicates that dairy products may be used in a weight maintenance diet without contributing to weight gain compared to diets that are low in dairy products.

## Competing interests

MBZ and JED have received grants from the National Dairy Council. MBZ holds patents covering uses of dietary calcium in weight management.

## Authors' contributions

MBZ and JED participated in the design, interpreted the results and helped draft the manuscript. DKS participated in the design and assisted with interpretation of the results and manuscript preparation. MSM participated in the study design and data analysis. GCW assisted with the data analysis and manuscript preparation. ELW and DM-H assisted with analysis and interpretation of the nutritional data and manuscript preparation. JR assisted with analysis of the clinical data and manuscript preparation. BB assisted with data acquisition and manuscript preparation. XS assisted with the design and interpretation of results. RAW assisted with the analysis and interpretation of the physical activity data and manuscript preparation.
